# Variation de l'albuminémie au cours de la malnutrition protéino-energétique dans une zone urbano-rurale congolaise

**DOI:** 10.11604/pamj.2015.20.299.5794

**Published:** 2015-03-26

**Authors:** Aimée Mudekereza Musimwa, Gray Wakamb Kanteng, Gayllord Nkashama Mutoke, Kristen Numbe Okito, Mick Ya Pongombo Shongo, Oscar Numbi Luboya

**Affiliations:** 1 Université de Lubumbashi, Département de Pédiatrie, Cliniques Universitaires de Lubumbashi, Congo

**Keywords:** Malnutrition, albuminémie, enfant, Lubumbashi, Malnutrition, albumin, infant, Lubumbashi

## Abstract

**Introduction:**

La malnutrition est à ce jour un problème de santé publique majeur, notamment dans les pays en voie de développement. Le diagnostic est fait cliniquement, mais l'intérêt de certains dosages paracliniques ont leur importance pour en évaluer la gravité ou faciliter un dépistage précoce, notamment de l'albuminémie. Cette étude a eu pour objectif de déterminer la variation de l'albuminémie au cours de la malnutrition protéino-calorique de l'enfant et de déterminer les facteurs associés.

**Méthodes:**

Il s'agit d'une étude descriptive transversale, effectuée prospectivement de juillet 2013 à mars 2014. 154 cas ont été colligés, par échantillonnage de convenance, avec un dépistage actif des enfants malnutris.

**Résultats:**

72,7% d'enfants avaient un taux normal d'albuminémie, ce taux bas étant pour la plupart lié à un état inflammatoire et/ou infectieux au cours de la malnutrition. Le taux d'albuminémie a un lien étroit avec l’état nutritionnel, chez le malnutri chronique, l’émacié et chez ceux présentant un déficit pondéral avec respectivement 18,3%; 24,0% et 30,4% d'enfants qui ont présenté un taux bas en albumine plasmatique. Cette hypo albuminémie a été retrouvé chez les malnutris avec ou sans œdèmes. 30 enfants ont présentés des œdèmes et 63% avaient un taux bas d'albumine sérique; contre 124 enfants qui n'ont pas présentés des œdèmes et 18,3% ont présenté un taux bas en albumine sérique.

**Conclusion:**

La malnutrition est une maladie dont les perturbations impliquent celle de l'albuminémie. Les variations de l'albuminémie sont statistiquement associées au tableau clinique.

## Introduction

La malnutrition est définie selon l'OMS comme étant un état pathologique résultant de la carence ou de l'excès relatif ou absolu d'un ou de plusieurs nutriments essentiels [1]. Elle joue un rôle dans au moins la moitié de décès des enfants dans le monde, ce qui est plus que n'importe quelle maladie infectieuse. Cliniquement, la malnutrition est décelable notamment par l’évaluation des paramètres anthropométriques. Néanmoins les analyses biochimiques ou physiologiques permettent d'en déterminer exactement la portée [1]. S'il n'existe aucun marqueur biologique spécifique de la malnutrition, l'utilisation de l'albumine et de la pré-albumine restent à ce jour appropriées pour une appréciation biologique de la malnutrition. Le taux d'albumine inférieur à 3,5g/l augmente le taux de la morbidité [2]. En rapport avec les variations de l'albumine au cours de la malnutrition, une étude réalisée en Côte d'Ivoire chez des enfants entre 0 et 5 ans a révélé que l'albumine constitue un indicateur peu fiable, car elle n'est diminuée que dans les formes graves [3]. Une étude effectuée sur la malnutrition protéino-énergétique aiguë de l'enfant camerounais a démontré que la protidémie était anormalement basse chez les enfants atteints de Kwashiorkor ou de kwashiorkor-marasme [4]. Une autre étude a été réalisée en RDC, au Kivu central sur les aspects cliniques et biologiques de la malnutrition protéino-calorique infantile, chez des sujets soignés au centre de nutrition de l'IRSAC, depuis 1959 jusque début 1965. Cette étude a prouvé que le taux de protidémie et albuminémie sont incontestablement très bas surtout en ce qui concerne l'albumine [5]. La présente étude a pour objectifs de déterminer le taux d'albumine plasmatique en cas de malnutrition aigüe de l'enfant, notamment dans les formes graves, ainsi que de décrire les manifestations cliniques associées à cette variation chez le malnutri.

## Méthodes

**Cadre de travail:** Notre travail a été effectué dans la zone urbaine et péri urbaine de Lubumbashi, au sud-est de la République Démocratique du Congo.

**Type et population d’étude:** Nous avons effectué une étude descriptive transversale, sur un mode prospectif, durant la période allant de juillet 2013 à mars 2014. Notre étude a porté sur les enfants de moins de 5 ans, traités pour malnutrition aiguë, en zone urbaine et péri urbaine de la ville de Lubumbashi.

**Méthode de collecte de données:** La récolte des données s'est déroulée en deux temps. Dans le premier temps, au sein de quatre structures hospitalières localisées dans la zone urbaine de la ville de Lubumbashi où sont organisées des unités de réhabilitation nutritionnelle pour enfants malnutris: il s'agit de l'hôpital Général de Référence Jason Sendwe, l'hôpital Général de Référence Kisanga, l'hôpital Militaire Camp Vangu, l'hôpital de Référence Mamba 2. En deuxième lieu, la récolte s'est faite en milieu rural, dans le village Kawama situé à 30 km de la ville de Lubumbashi, par dépistage actif des enfants malnutris au sein la population par la méthode de porte à porte.

**Echantillonnage:** Notre échantillonnage était de convenance, comprenant 154 cas répertoriés au sein des centres de réhabilitation nutritionnels des hôpitaux suivants: Sendwe, camps Vangu, Kisanga, Mamba 2; mais aussi dans un milieu extrahospitalier (village Kawama), selon les critères d'inclusion ici-bas. Ont été inclus dans notre étude: Tout enfant avec un âge compris entre 6-59 mois, nouvellement admis pour malnutrition aigüe dans l'un des centres pré cités et durant la période choisie pour l’étude, soit une période de 9 mois; Le diagnostic de la malnutrition a été posé de deux façons: de manière passive à l'admission dans les centres avec comme critère pour la MAM un indice P/T > ou = -3Z score et < -2Z score et pour la MAS un indice P/T < -3Z score; puis la présence ou l'absence d’œdèmes qui nous a permis de faire la différence entre le marasme et le kwashiorkor; De manière active de porte en porte en zone extrahospitalière (village Kawama), le diagnostic de la MAM a été fait lorsque le périmètre brachial été supérieur ou égal à 11,5cm et inférieur à 12,5 cm; quand il été inférieur à 11,5 cm, on a diagnostiqué la MAS. Ont été exclu de notre travail: les enfants dont les parents n'ont pas présenté leur accord; les enfants ayant été transfusés quelques jours avant le prélèvement et tout enfant ayant debuté le traitement.

**Technique de récolte:** Voici les différents matériels nous ayant permis à réaliser notre récolte: une fiche de récolte contenant un questionnaire rempli par interview des parents; une toise, chez les enfants avec un âge > 24mois en position debout, on a noté la taille en cm, et chez les enfants avec un âge < 24mois en position couchée, la longueur a été notée en cm; un mètre ruban, pour la mesure du périmètre brachial, au niveau du bras à mi-distance entre l'olécrane et l'acromion. Et aussi pour la mesure du périmètre crânien; une balance SALTER, pour la mesure du poids; une balance pèse personne, quand nous n'avions pas de balance pèse bébé. La mère était pesée seule d'abord, puis avec son enfant et la soustraction des deux poids obtenus était faite après; thermomètre électronique pour la prise de la température, en le plaçant dans le creux axillaire; prélèvements du sang veineux effectués sur chaque enfant à l'aide d'une seringue de 5cc et conservé dans des tubes contenant du citrate lesquels tubes étaient acheminés le même jour au laboratoire médical des Cliniques Universitaire de Lubumbashi; une glacière pour y mettre nos prélèvements sanguins; de l'ouate, l'alcool dénaturé, et un garrot, pour le prélèvement d'environ 1cc de sang veineux.

**Analyses statistiques:** La détermination de l’état nutritionnel s'est faite sur base des indicateurs suivants: poids, taille, âge, z-score. Ce dernier était calculé à l'aide du logiciel Ena version 2007 (Emergency Nutrition Assessment) et a été classifié de la manière suivante: état nutritionnel normal (Z-score supérieur ou égal à -1,00), malnutrition légère (Z-score compris entre -2,00 à -1,01), malnutrition modérée (Z-score compris entre -3,00 à -2,01) et malnutrition sévère (Z-score inférieur à -3,00) (USAID. Evaluation de la situation nutritionnelle dans les situations d'urgence. Juin 2006’ > USAID. Evaluation de la situation nutritionnelle dans les situations d'urgence. Juin 2006, www.fantaproject.org).

**Considérations éthiques:** Le protocole du travail a été soumis et approuvé au Département de Pédiatrie des Cliniques Universitaires de Lubumbashi. Nos prélèvements ont été effectués sous le consentement verbal libre et éclairé des parents de chaque enfant, après une explication brève du but de notre étude.

**Tableau 1:** Répartition des cas selon le taux d'albumine sérique

Taux d'albumine (en g/dl)	Effectif	Pourcentage
<2,5	14	9,1
2,5 -3,0	15	9,7
3,0 -3,4	13	8,4
≥3,5	112	72,7
Total	154	100

(Taux de référence normale d'albumine: >3,5g/dl)

**Tableau 2:** Rapport entre l'albumine sérique et la présence d’œdèmes

Œdèmes Albumine (g/dl)	Présents			Absents	Total	P	OR [IC95%]
	N	%	N	%			
<2,5	9	30,0	5	4,0	14	0,0000	16,52 [4,69-58,14]
2,5 -3,0	6	20,0	9	7,3	15	0,0048	6,12 [1,83-20,44]
3,0 -3,4	4	13,3	9	7,3	13	0,0505	4,01 [0,77-17,69]
≥3,5	11	36,7	101	81,5	112	-	1
Total	30	100	124	100	154		

**Tableau 3:** Relation entre Z-score Poids pour âge et albumine

Z-score	<-2 ET		≥-2 ET		Total		p	OR [IC95%]
Albumine	n	%	n	%	n	%		
<3,0 g/dl	24	24,0	5	9,3	29	18,8	0,0437	3,09 (1,10-8,65)
≥3,0 g/dl	76	76,0	49	90,7	125	81,2		
Total	100	100	54	100	154	100		

## Résultats

L’âge moyen des enfants est de 24,8±13,8 mois; les extrêmes sont de 6 et 59 mois. Près de la moitié des enfants avaient un âge compris entre 12 et 23 mois. Le sexe féminin est le plus prédominant avec 54,5% (84/154) des cas soit un sexe ratio de 1,2. La plupart des patients ont une alimentation usuelle identique à celle du repas familial (66,9%); 97/154 enfants avaient déjà arrêté d’être nourris au sein. La durée moyenne d'allaitement est de 15,7±7,0 mois; les extrêmes sont de 0 et 36 mois. La médiane est de 16 mois et le mode de 24 mois. Soixante-sept pourcent de nos patients ont été allaité pendant plus de 12 mois. L’âge moyen lors de la diversification alimentaire chez nos patients est de 4,5±3,9 mois; les extrêmes sont de 1 et 30 mois. La médiane et le mode sont de 3 mois. Septante-sept pourcent de nos patients ont eu une diversification alimentaire précoce (Tableau 4). En rapport avec le rapport Z-score Taille pour âge et l'albumine, La moyenne du taux d'albumine est de 3,6±0,9 g/dl chez les enfants malnutris (z-score <-2 ET) allant de 1,9 à 6,1 g/dl alors qu'elle est de 4,1±0,9 g/dl chez les enfants avec z-score ≥-2 ET allant de 1,4 à 6,3 g/dl; la comparaison de ces deux moyennes donne une différence statistiquement significative (t = 3,27; p = 0,0013). En comparant les proportions de déficit grave en albumine chez les malnutris aigus et chez les non malnutris aigus, le test de Chi carré corrigé montre une différence statistiquement significative (30,4% versus 12,2%; p = 0,0107) avec un risque multiplié par 3 fois pour que l'amaigrissement soit associé à un déficit grave en albumine (OR: 3,12; IC95%: 1,36-7,16) (Tableau 5).

**Tableau 4:** Relation entre Z-score Taille pour âge et albumine

Z-score	<-2 ET		≥-2 ET		Total		p	OR [IC95%]
Albumine	n	%	n	%	n	%		
<3,0 g/dl	23	18,3	6	21,4	29	18,8	0,9033	0,81 [0,29-2,24]
≥3,0 g/dl	103	81,8	22	78,6	125	81,2		
Total	126	100	28	100	154	100		

**Tableau 5:** Relation entre Z-score Poids pour taille et albumine

Z-score	<-2 ET		≥-2 ET		Total		p	OR [IC95%]
Albumine	n	%	n	%	n	%		
<3,0 g/dl	17	30,4	12	12,2	29	18,8	0,0107	3,12 [1,36-7,16]
≥3,0 g/dl	39	69,6	86	87,8	125	81,2		
Total	56	100	98	100	154	100		

## Discussion

### Aspect nutritionnel des enfants

**a. Type d'alimentation:** Dans notre étude, il ressort que la plupart d'enfants étaient nourris au plat familial avec un taux d'environ 66,9%. La durée moyenne d'allaitement étant de 15,7 +/- 7 mois. 67% d'enfants ont été allaités au-delà de 12 mois et 9% d'enfants ont été allaités endéans une période inférieur à 6 mois. 77% d'enfants ont eu une diversification alimentaire précoce c'est-à-dire à un âge inférieur à 6 mois. Sur ce point, la plupart des études rencontrées ont montrent une association entre une alimentation inadaptée et la dénutrition des enfants. Par exemple, une étude effectuée en Inde a révélé que la prévalence des enfants non allaités été de 13%, celle des enfants avec une alimentation mixte été de 6% et celle des enfants avec un allaitement exclusif été de 9,4% [6]. Une étude effectuée au Burkina Faso en 2003 sur 8628 enfants recrutés qui a portée sur l'allaitement et l’état nutritionnel de l'enfant. Cette étude a démontré que les pratiques d'alimentation constituent un facteur déterminant de l’état nutritionnel de l'enfant. La majorité des enfants ont été allaité, à environ 98%, et cette pratique s'est poursuivi jusqu’à environ 20-23 mois ou on note un taux d'environ 81% d'enfants encore allaité. L'allaitement a été exclusif pour 18% enfants jusqu’à 4-5 mois. Rien que 48% d'enfants bénéficient d'une alimentation complémentaire outre le lait maternel. A un âge de 16-19 mois, un taux de 83% d'enfants ont reçus une alimentation complémentaire en plus de l'allaitement [7]. Il a été noté que les enfants dont les mères vivent dans un milieu rural ont été allaité pendant longtemps par rapport à ceux dont les mères sont venues des milieux urbains; cette répartition a été aussi faite selon le niveau d'instruction de la mère. Les mères plus instruites allaitent moins leur enfant que celles moins instruites. D'où, nous constatons que type d'alimentation de l'enfant est étroitement lié à son état nutritionnel, un enfant recevant un aliment complémentaire avant l’âge de 6 mois est plus exposé à contracter diverses maladies infectieuses et elle diminue la prise de lait par l'enfant, et dans les populations pauvres, les aliments de compléments sont souvent pauvres en nutriment, ce qui expose l'enfant à développer une malnutrition chronique ou aigue.

**b. Aspect nutritionnel selon le Z-score:** Dans notre étude le Z-score P/A avec une moyenne de -2,8 + - 1,6; on a noté une insuffisance pondérale sévère chez 42,9% d'enfants et les 22,1% ont présenté une insuffisance pondérale modérée. La répartition selon le Z-score T/A avec une moyenne de -3,7 +- 1,8 a noté un taux de 76,1% des patients présentant une malnutrition chronique. La répartition selon le Z-score P/T avec une moyenne de -1,0 +- 2,4 a noté un taux de 17,5% d'enfants présentant une malnutrition aigue et un taux de 18,8% d'enfants avec une malnutrition modérée. Une étude effectuée en Ethiopie sur 180 ménages chez des enfants de 6-59 mois. Cette étude a explorée la prévalence de la malnutrition en rapport avec les caractères sociodémographiques. Elle a évaluée le Z- score T/A, P/T et P/A pour mesurer l'ampleur du retard de croissance, de l’émaciation et de l'insuffisance pondérale. Les résultats ont révélé que la prévalence d'un long déséquilibre nutritionnel est l'indicateur d'un retard de croissance et un taux de 67,8%; et un déséquilibre nutritionnel à court terme, note une émaciation avec un taux de 12,8% et une insuffisance pondérale de 46,1% [8]. Une enquête intéressant 400 enfants de moins de 5ans, dans le district sanitaire de Khombole du 17 au 25 avril 1997 dans le but d’évaluer la prévalence de la malnutrition et les facteurs de risque qui lui sont associés. L’émaciation intéresse 8% d'enfant et le retard de croissance statural 34,7%, 12,7% d'enfants présentent une association de l’émaciation et du retard statural. La malnutrition aigue prédomine dans la tranche d’âge de 7 à 47 mois et son pic est situé entre 12 et 23 mois (17,4%); la malnutrition chronique quant à elle, elle se voit notable entre l’âge de 7 et 11 mois avec un taux de 20,5% et intéresse près de la moitié d'enfants au-delà de 12mois [9].

**Relations entre l’état nutritionnel et le taux d'albuminémie** Notre étude a rapporté qu'un taux de 72,7% d'enfants présentait un taux normal d'albuminémie. La comparaison du taux d'albuminémie entre les enfants présentant des œdèmes et ceux n'en présentant pas, cela montre une différence statistiquement significative; lorsqu'il s'agit d'un déficit sévère et d'un déficit grave respectivement 30% versus 4% et 20% versus 7,3%. En ce qui concerne l’état nutritionnel, nos résultats montre une différence statistiquement significative pour le Z-score P/A. en comparant la moyenne du taux d'albuminémie chez les enfants malnutris Z-score < -2 ET, et ceux avec un Z-score > ou = -2 ET. Il ressort que, un taux de 24% d'enfants insuffisants pondéraux présente un déficit marqué en albumine (< 3,0g/dl) contre un taux de 9,3% chez les non insuffisants pondéraux. L'analyse statistique donne une différence statistiquement significative de ces deux proportions, montrant que l'insuffisance pondérale est indexée d'un risque 3 fois d’être associée d'un déficit grave en albumine. En ce qui concerne le Z-score T/A, la comparaison des deux moyennes chez les enfants malnutris avec un Z-score < -2ET et ceux avec un Z-score ≥ -2ET, ne donne pas de différence statistiquement significative. 18,8% d'enfants malnutris chroniques avaient un déficit grave en albumine, contre 21,4% d'enfants non malnutris chroniques. Parlant du Z-score P/T, la comparaison de ces deux moyennes donne une différence statistiquement significative. En comparant la proportion de déficit grave en albumine chez les malnutris aigus et chez les non malnutris aigus, 30,4% versus 12,2% avec un risque multiplié par 3 fois pour que l'amaigrissement soit associé à un déficit grave en albumine. Une étude prospective portant sur l’évaluation de l’état nutritionnel de l'enfant ivoirien âgé de moins de 5ans outre les mesures anthropométriques, à déterminer l'albumine, transferrine, rétinol-binding protein et la thyroxine-binding préalbumine. L’état infectieux et/ou inflammatoire a été exploré par le dosage de la protéine C réactive et de l'al-glycoprotéine acide (al-GPA). L’étude a portée sur 56 enfants malnutris comparativement à 54 enfants normonutris de même âge et présumés sains. Les résultats ont montré une forte prévalence des états inflammatoires et/ou infectieux au cours de la malnutrition: 43% d’élévation simultanée de la CRP et de l'al-GRP ont été observés. L'incidence des perturbations du profil protéique et des infections associées est beaucoup plus importante dans la malnutrition sévère au cours de laquelle il a été noté u, index pronostique inflammatoire et nutritionnel très élevé [10]. Une autre étude effectuée, qui a portée sur l'effet de la malnutrition mineur et modérée sur la protéine immunitaire, inflammatoire et nutritionnelle chez l'enfant en cote d'ivoire, en 2010. 142 enfants recrutés dont 42 malnutris, 30 mineurs et 12 modérés. Les résultats ont montrés que l'albumine a été abaissée (p 0,05) au cours de la malnutrition modérée et mineure en comparaison aux sujets normonutris. Il a été noté une élévation de la CRP dans les deux formes de malnutrition. Cette étude a montré que les malnutritions mineures et modérées chez l'enfant sont accompagnées toujours de processus inflammatoire et d'une consommation protéique notamment de l'albumine [11]. Une étude effectuée en côte d'ivoire sur 56 (13 malnutris mineur, 23 malnutris moyens et 20 malnutris sévère) jeunes enfants âgés de moins de 5 ans, qui a évalué les marqueurs protéiques dans les états de malnutrition. Dans cette études, 4 protéines viscérales (albumine, transferrine, rétinol-binding protein et la thyroxine binding-protein) ont été évalué et deux protéines de l'inflammation (la protéine-c-réactive et l'al-glycoprotéine acide). Les résultats ont montré que la thyroxine binding protéine est basse même dans les formes mineures alors que l'albumine a été noté basse dans les formes sévères. Une diminution des 4 protéines de la nutrition est observée dans les formes sévères type kwashiorkor ou marasme. Et il a été constaté que les protéines inflammatoires étaient fortement augmentées au détriment des protéines nutritionnelles [12]. Dans notre étude, sur 30 patients ayant présentés des œdèmes, 19 patients ont eu une association avec une hypo albuminémie sérique. Et sur 124 patients n'ayant pas présentés d’œdèmes, 20 patients ont eu un taux d'albuminémie inférieur au taux normal.

## Conclusion

Cette étude avait pour objectif principal d’étudier la variation de l'albuminémie au cours de la malnutrition. Le type d'alimentation de l'enfant est étroitement lié à son état nutritionnel. Ainsi, un enfant recevant un aliment complémentaire avant l’âge de 6 mois est plus exposé à faire une malnutrition. La majorité des enfants souffrant de malnutrition sont issus des ménages à conditions sociodémographiques défavorables dont la pauvreté, la faible éducation et le faible niveau scolaire des parents, surtout des mères, en sont la base. Il a été noté un déficit en albumine sérique chez la plupart des malnutris aigus comme chroniques, avec un risque élevé pour l'amaigrissement qu'il soit associé à un déficit grave en albumine sérique. Cette baisse d'albumine sérique était pour la plupart liée à un état inflammatoire et/ou infectieux au cours de la malnutrition.
